# Supplementary material for: Quantifying Parkinson’s disease severity using mobile wearable devices and machine learning: the ParkApp pilot study protocol
Source: BMJ Open. 2023 Dec 28;13(12):e077766. doi: 10.1136/bmjopen-2023-077766 (PMC10759062; doi:10.1136/bmjopen-2023-077766)
Supplement: Supplementary data [file bmjopen-2023-077766supp001.pdf]

Appendix 1

Daily sleep questionnaire.

Intro:
The following questions are related to the quality of your sleep last night. The answers provided here will be compared with the activity measured by the activity band.

Questions:
1) At what time did you go to sleep yesterday?

2) At what time did you wake up?

3) How did you perceive the quality of sleep last night?
Excellent - Good - Fair – Poor

4) Was falling asleep difficult?
No - Somewhat - Fairly – Very

5) Did you lie awake for long periods during the night?
No - Some - Fairly much - Very much

6) Did you suffer from interruptions?
No - Some - Fairly much - Very much

Appendix 2

The interview will follow a semi-structured format with questions based on usability, acceptance and trust models. One person will interview the patient either in the clinic or over the phone. Interviews will be audio recorded and notes will be taken. Interviews will last no longer than 30 minutes.
The following are example questions which the interviewers may adapt depending on the context. To keep the interview time limited, 2 questions for theme will be chosen and, depending on the provided answers, more questions may be asked to further elaborate some concepts.

Theme: Participation

1. Why did you join this study?
2. Have you appreciated being part of a research study, or has it been a bother to you? Why do you feel this way?
3. Would you be interested in future participation in research, if there was an opportunity to do so? Why?
4. Would you like to keep using the app to participate in other studies?

Theme: Onboarding, understanding and engagement

1. How long did it take you to start using Mobistudy?
2. Did you encounter any technical issue?
3. Were there parts (e.g. tests or menus) that were hard to know if you did the right thing with? Which? Why?
4. Are there tests (if so, which) that you still have questions on how they work, or do you understand all of them now?
5. Any test that you were particularly interested in the results from? Why?
6. Is there anything we could add or remove from the app to make it more interesting for you?

Theme: Impact (conventional clinical practice vs app use)

1. Are there parts of your life that are affected by your PD which you are not reporting in the traditional clinical meetings?
2. Are there parts of your life that are affected by your PD which you aren't reporting in the Mobistudy app?
3. Have you learned something new about your own PD during the research project? What? How?
4. How does this impact your day-to-day life?
5. Did the use of the app create any concern about your health?
6. Do you think using the app would improve the way your doctors keep your health under control?
7. Would you trust this technology to substitute your periodic appointment with your doctor?

Theme: Trust

1. Do you have any concern about your data being shared with parties that you do not trust?
2. What sort of parties do you consider untrustworthy?
3. Did you have any concern about being "observed" (even if by people you trust)?
4. Can you give me examples of apps you trust?
5. And apps you do not trust?
