# Supplementary material for: Quantifying Parkinson’s disease severity using mobile wearable devices and machine learning: the ParkApp pilot study protocol
Source: BMJ Open. 2023 Dec 28;13(12):e077766. doi: 10.1136/bmjopen-2023-077766 (PMC10759062; doi:10.1136/bmjopen-2023-077766)
Supplement: Author's manuscript [file bmjopen-2023-077766.draft_revisions.pdf]

BMJ Open is committed to open peer review. As part of this commitment we make the peer review history of every article we publish publicly available.

When an article is published we post the peer reviewers' comments and the authors' responses online. We also post the versions of the paper that were used during peer review. These are the versions that the peer review comments apply to.

The versions of the paper that follow are the versions that were submitted during the peer review process. They are not the versions of record or the final published versions. They should not be cited or distributed as the published version of this manuscript.

BMJ Open is an open access journal and the full, final, typeset and author-corrected version of record of the manuscript is available on our site with no access controls, subscription charges or pay-per-view fees (http://bmjopen.bmj.com).

If you have any questions on BMJ Open's open peer review process please email info.bmjopen@bmj.com

BMJ Open

Quantifying Parkinson's disease severity using mobile wearable devices and machine learning; The ParkApp pilot study protocol

Journal:	*BMJ Open*
Manuscript ID	bmjopen-2023-077766
Article Type:	Protocol
Date Submitted by the Author:	14-Jul-2023
Complete List of Authors:	Ymeri, Gent; Malmo universitet, DVMT<br>Salvi, Dario; Malmö University, DVMT<br>Olsson, Carl Magnus; Malmö universitet, DVMT<br>Wassenburg, Myrthe; Karolinska Institute, Department of Clinical Neuroscience; Academic Specialist Center Torsplan<br>Tsanas, Athanasios; University of Edinburgh<br>Svenningsson, Per; Karolinska Institute, Department of Clinical Neuroscience; Academic Specialist Center Torsplan
Keywords:	Parkinson-s disease < NEUROLOGY, Health informatics < BIOTECHNOLOGY & BIOINFORMATICS, Telemedicine < BIOTECHNOLOGY & BIOINFORMATICS

SCHOLARONE™
Manuscripts

Quantifying Parkinson's disease severity using mobile wearable devices and machine learning; The ParkApp pilot study protocol

For peer review only

Gent Ymeri 1, Dario Salvi 1, Carl Magnus Olsson 1, Myrthe Wassenburg 2,3, Athanasios Tsanas 4,5, and Per Svenningsson 2,3

1 Internet of Things and People research center, Department of Computer Science and Media Technology (DVMT), Malmö University, Sweden
2 Department of Clinical Neuroscience, Karolinska Institute, Sweden
3 Center for Neurology, Academic Specialist Center Torsplan, Region Stockholm, Sweden
4 Usher Institute, Edinburgh Medical School, The University of Edinburgh, United Kingdom
5 Alan Turing Institute, London, United Kingdom

1 firstname.lastname@mau.se
2, 3 firstname.lastname@ki.se
4, 5 firstname.lastname@ed.ac.uk

Corresponding author:
Gent Ymeri; Bassänggatan 2, 211 19, Malmö, Sweden; gent.ymeri@mau.se; +46 40-665 71 71

Abstract

Introduction: the clinical assessment of Parkinson's disease (PD) symptoms can present reliability issues and, with visits typically spaced apart 6 months, can hardly capture their frequent variability. Smartphones and smartwatches along with signal processing and machine learning can facilitate frequent, remote, reliable, and objective assessments of PD from patients' homes.

Aim: To investigate the feasibility, compliance, and user experience of passively and actively measuring symptoms from home environments using data from sensors embedded in smartphones and a wrist-wearable device.

Methods and analysis: In an ongoing clinical feasibility study, participants with a confirmed PD diagnosis are being recruited. Participants perform activity tests, including Timed Up and Go (TUG), tremor, finger tapping, drawing, and vocalization, once a week for two months using the Mobistudy smartphone app in their homes. Concurrently, participants wear the

GENEActiv wrist device for 28 days to measure actigraphy continuously. In addition to using sensors, participants complete the Beck's Depression Inventory, NMSQuest and PDQ-8 questionnaires at baseline, at 1 month and at the end of the study. Sleep disorders are assessed through the Parkinson's Disease Sleep Scale-2 questionnaire (weekly) and a custom sleep quality daily questionnaire. User experience questionnaires, TAM and uMARS are delivered at 1 month. Clinical assessment (MDS-UPDRS) is performed at enrollment and the two-month follow-up visit. During visits, a TUG test is performed using the smartphone and the G-Walk motion sensor as reference device.

Signal processing and machine learning techniques will be employed to analyze the data collected from Mobistudy app and the GENEActiv and correlate them with the MDS-UPDRS. Compliance and user aspects will be informing the long-term feasibility.

Ethics and Dissemination: The study received ethical approval by the Swedish Ethical Review Authority (Etikprövningsmyndigheten), with application number (2022-02885-01). Results will be reported in peer-reviewed journals and conferences. Results will be shared with the study participants.

Keywords: Parkinson’s Disease, Health Informatics, Telemedicine

Wordcount: 3113

Strengths and limitations of this study

- This study utilizes a mobile and wearable device to conveniently, non-invasively, passively, and actively quantify symptoms at home in a user-friendly manner.
- The study combines objective data collected across multiple tests, to evaluate the frequency and severity of motor and non-motor symptoms.
- The study aims at identifying the link between data collected at home and conventional clinical scales, potentially enhancing the remote monitoring and management of Parkinson's disease.
- This study excludes participants with severe symptoms who are unable to use smartphones, which may result in a less diverse sample and limit the generalizability of the findings.
- Data analysis is dependent on the quantity and variability of collected data; success of the study relies on participants’ compliance with the intensive data collection regime.

Introduction

Parkinson’s disease (PD) is a heterogeneous neurodegenerative disorder characterized by third cardinal symptoms: bradykinesia, resting tremor and musculoskeletal rigidity as well as several non-motor symptoms including cognitive alterations, anxiety, depression, hallucinations, pain, speech issues, sleep disorders along with autonomic disorders such as bladder and bowel problems and orthostatic hypotension (1). PD is the second most common neurodegenerative disorder, with a worldwide prevalence estimated to reach 13 million cases by 2040 (2). The diagnosis and assessment of PD are mainly based on clinical findings such as history and physical examinations (3). The most used and recommended

For peer review only

rating scales to measure PD disease severity, progression and functional disability are the Hoehn and Yahr (H&Y) staging scale and the MDS-Unified Parkinson Disease Rating Scale (MDS-UPDRS)(4,5). The MDS-UPDRS is usually performed twice a year during clinic appointments to track disease progression. Both scales can present reliability issues, use ordinal scores and do not track disease burden in a home environment at regular intervals (6).

To complement in-clinic examinations, patients may be asked to record their symptoms in diaries at home; however, these diaries often lack the necessary accuracy (7) needed to effectively supplement clinical assessments and are prone to recall bias. Smartphones offer a more convenient alternative to pen and paper, allowing for more efficiency and accuracy while minimizing such biases (8). Nevertheless, self-reports alone are insufficient to capture the full extent of symptoms, and additional technologies have been demonstrated to provide significant benefits across a range of applications (9). Consequently, there is a need for systems that allow PD patients to be conveniently, reliably, objectively, and longitudinally monitored in their home environments.

Accelerometers, particularly those embedded in mobile phones and smartwatches, have recently been explored as cost-effective, user-friendly solutions to monitor PD symptoms, and facilitating better follow-up, care and improved patient management (10–12). As an example, Lopez-Blanco and co-workers (13) explore the gyroscope embedded in smartwatches to analyze rest tremor in PD patients. Their results show that utilizing smartwatches for assessing rest tremor can correlate with clinical score and is well accepted from users. Czech and co-workers (14) investigate wearable devices to measure gait in PD. Their results prove that measuring gait in PD can be performed reliably with a single accelerometer. However, their study uses a lumbar-mounted accelerometer and does not investigate common wearable devices such as smartwatches or smartphones. Some research projects extend the range of monitored symptoms, such as in (15) where the authors investigate the quantification of dexterity in PD through a smartphone-based system. In this paper, authors incorporate finger tapping and drawing tests in a clinical trial including 19 PD participants and 22 healthy controls. Their results show weak to moderate correlations between UPDRS items and smartphone-based ratings; however, this study was performed in a clinical environment and does not show that the same quality of data can be obtained at home. In addition, the more recent mPower study (16) explores assessment of PD utilizing a smartphone application in home environments. Recruitment of participants in this study was performed online which did not guarantee that all the participants were clinically diagnosed with PD. This study incorporates a number of tasks to gather data with the participants' mobile phones, including finger tapping, vocalization exercises, walk, balance and memory tests. Their results show that the finger tapping task was more predictive for the self-reported status of PD. A smartphone app, cloudUPDRS app, is also introduced in (17) which also includes a number of data collection activities such as reaction, tremor and walking tests, and a wellbeing test, but excluding voice or cognition tests.

Despite the existence of various digital health tools for PD monitoring, these typically focus on a single measurement modality, or the recruitment of the participants does not generalize the results for at-home assessments. A multimodal, user-friendly, and affordable

For peer review only

solution for home-based continuous monitoring of PD could provide significant advantages for both patients and clinicians.

Considering this, the primary aim of this study is to determine the feasibility of reliably measuring PD symptoms using mobile phones and wearable devices at home and to provide a more holistic overview of symptomatology compared to what has been attempted in previous studies. The study is supported by the development of a novel, multi-modal symptoms tracker system, which both passively and actively quantifies motor symptoms such as bradykinesia, gait impairment, tremor, voice disorders, as well as non-motor symptoms like depression, and sleep disturbances.

Methods and Analysis

Study design and participants

This clinical feasibility study involves the recruitment of 40 participants with clinically diagnosed PD at the Center for neurology, Academic Specialist Center Torsplan (ASCT), Stockholm Health Services, Region Stockholm.

Participants are recruited based on the inclusion and exclusion criteria summarized in Table 1. Guided by similar studies that have used between 10-50 participants (18–20), the goal of this study is towards hypothesis generation and assessing feasibility. Efforts are made to recruit participants with a diverse range of PD symptoms while avoiding over-representation of one sex or age category. Given the limited number of participants and the exploratory nature of this study, no specific software is used for randomization; an ad-hoc approach is adopted depending on the circumstances. Recruitment started in the fourth quarter of 2022, with plans to complete data collection in the fourth quarter of 2023.

Table 1: Inclusion/ exclusion criteria

Inclusion criteria	*Exclusion criteria*
Must be diagnosed with Parkinson's Disease and under treatment at Karolinska University Hospital or Academic Specialist Center Torsplan	Not able to use a smartphone
Must have mild or moderate symptoms	Unable to read and understand Swedish
Must own a smartphone, Android or iPhone	Severe symptoms (H&Y 3 and above)

Outcomes

The research questions that this study addresses are:

A. Is it possible to link multimodal data collected by mobile phones and wearable devices to provide a more holistic understanding of Parkinson's symptoms?
B. What extracted information/ features from raw data are predictive of MDS-UPDRS scores using data collected by mobile phones and wearables?
C. Are mobile phones and wearable devices usable and accepted for data collection?

For peer review only

These research questions entail collecting data from devices at participants' home and reference data in-clinic and developing algorithms to associate the MDS-UPDRS score from the data collected at home. The questions also imply measuring compliance to the data regime protocol, usability of the technology and long-term acceptance.

Data collection

Data from participants are collected through two clinic visits - at baseline and after two months - as well as in between visits by using a mobile phone app and a wearable device in home environment. The mobile phone app used in this study is Mobistudy (21), a free and open-source app designed for clinical research. The app prompts users to perform scheduled tasks during their two-month participation in the study, as outlined in Table 2. Tasks include answering questionnaires and performing motor tests. The motor tests are prompted to be performed twice: once prior to medication intake and again post-intake to measure ON and OFF states. While there is no designated time of day for these tests, participants are instructed to consistently follow this pre- and post-medication schedule.

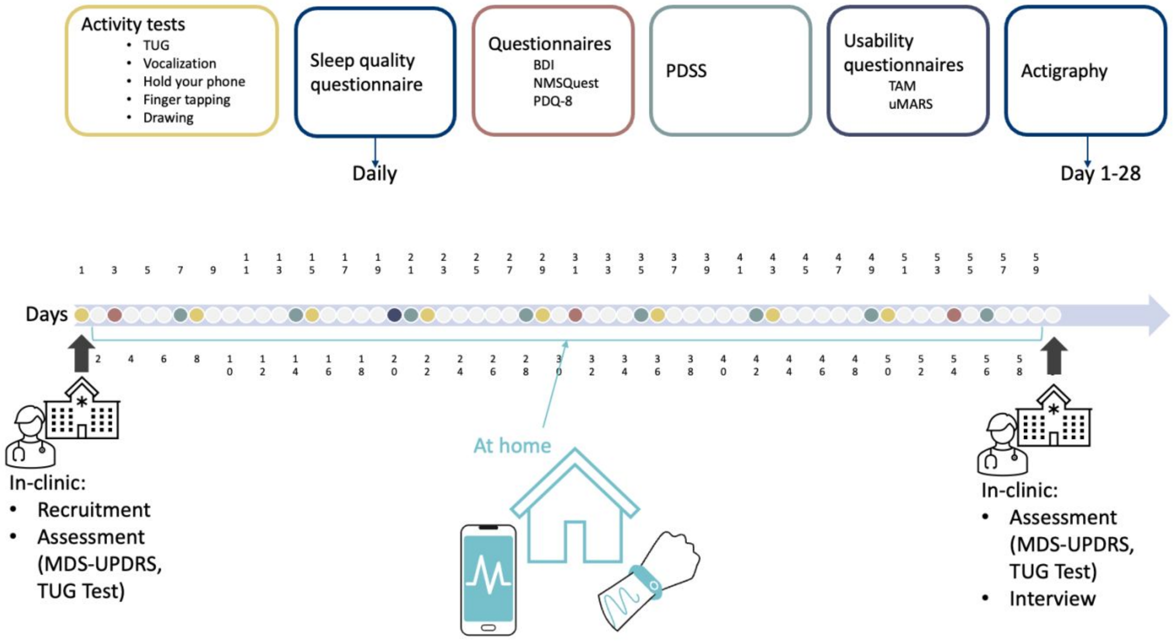

Figure 1 Timeline of the study depicting assessments, tests and questionnaires, when and where they take place.

The tasks included in the Mobistudy app for this study are:

- Questionnaires:
    - Beck's depression inventory-II (BDI-II) (22). This is used to quantify depression.
    - Parkinson's disease sleep scale (PDSS-2) (23). Used to quantify sleep disorder.
    - NMSQuest (24). Measures non-motor symptoms.

- PDQ-8 (25). Short scale to assess motor and non-motor symptoms in daily life.
- Sleep quality questionnaire. Based on (26), is a custom daily short questionnaire to assess sleep quality (full questions list in Appendix).
- User Version of the Mobile Application Rating Scale (uMARS) (27). Measures usability and the quality of the app.
- Technology Acceptance Model (TAM) (28). Estimates long-term usage of the app.

- Timed up and go test (TUG test): Participants are instructed to stand up from a chair, walk 3 meters, turn around, return to the chair, and sit down. The mobile phone, secured in a waist belt around the hip, records data from inertial sensors (accelerometer and gyroscope) and estimates the total time required to complete the task.
- Vocalization test: Participants record their voice during a vocalization exercise guided by the app. The exercise consists of pronouncing the sustained vowels “a”, “i” and “u” for as long as possible.
- Hold the phone test (HTP test): Participants are instructed to hold the phone for 60 seconds while the phone records inertial sensors data (acceleration and orientation). The task is repeated for each hand in three positions: resting the hand holding the phone in the lap while sitting, extending the arm at shoulder’s height, and moving the arm from outstretched to touching the nose repeatedly.
- Finger tapping test (FT test): Participants are instructed to rapidly tap two buttons on the screen using the index and middle fingers.
- Drawing test: Participants are instructed to draw a square and a squared spiral on the screen by tracing a marked line with their index finger.

In addition to Mobistudy, a wrist-worn actigraphy device (GENEActiv, Activinsights, UK) is used to collect continuous three-dimensional acceleration data in free-living conditions. A gait analysis device (GWALK, BTS Bioengineering, Italy) is used to gather reference gait analysis data during the TUG test in clinic, at enrolment and conclusion of the study. The GENEActiv device is set to capture data at a sample rate of 25Hz. Whilst 10Hz is considered sufficient for wrist-based acceleration in day-to-day activities (29), this study adhers to the Nyquist theorem by using at least twice the maximum frequency of tremor (30).

Participants are approached during their regular visits at the ASCT clinic. If they give informed consent to participate, they receive an introduction to the study, instructions, a waist belt and an actigraphy device. The Mobistudy app is downloaded and installed on their smartphone, and participants create an account. A series of demo tests using Mobistudy is available for participants to practice and ensure understanding before the study starts. Each test includes in-app instructions. A TUG test, performed with the GWALK gait analysis reference device, and a full MDS-UPDRS assessment by a neurologist, are conducted during enrollment.
During the eight-week at-home period, participants complete tests and answer questionnaires using their phones and the Mobistudy app. Participants are contacted at day ten and at day 30 either via email or phone call to check compliance, the presence of any technical issues and asked for feedback on user-friendliness of the app.

For peer review only

Upon completing of the study, participants attend a second clinic visit, during which they perform a TUG test with GWALK, undergo a full MDS-UPDRS assessment and complete an interview. The semi-structured interview is designed to complement questions asked in the usability questionnaires and covers motivations for participation, usability, user friendliness, acceptance, perceptions on trust, and suggestions for improvements. A researcher conducts the interview with the participant either in the clinic or over the phone if consent is granted. Interviews are expected to last no more than 30 minutes and are audio recorded.

Table 2: The schedule for when activity tasks and questionnaires are performed by the recruited participants.

Activity tasks and Questionnaires	*Day after recruitment*							
Timed-up-and-go-test	1	8	15	22	29	36	43	50
Beck's depression inventory	3	31	54					
Parkinson's disease sleep scale	7	14	21	28	35	42	49	56
NMSQuest	3	31	54					
PDQ-8	3	31	54					
Vocalization test	1	8	15	22	29	36	43	50
Hold your phone test	1	8	15	22	29	36	43	50
Finger tapping test	1	8	15	22	29	36	43	50
Drawing test	1	8	15	22	29	36	43	50
Sleep quality questionnaire	Daily							
uMARS	20							
TAM	20							
Actigraphy	Continuously, recording at 25Hz until battery is depleted after 28 days							

Data Management

Clinical data, including patients' demographics (age, sex, year of diagnosis of PD, year of onset of first symptoms, deep brain stimulation device status, any PD-related surgery, medication regime and comorbidities) as well as in-clinic results (evaluated MDS-UPDRS and TUG test), are collected by the ASCT. These data are stored on an encrypted disk on a computer managed by the research team.

App-based data is collected using the Mobistudy platform (21). This includes participants' profiles - name, surname, date of birth, sex, height, weight, long-term diseases, long-term medications-, questionnaire responses and sensors data. Sensor's data includes acceleration and orientation during the TUG test and HTP test, voice recordings collected during the vocalization test, timestamps of taps during the finger tapping test and finger coordinates during the drawing test. Metadata including time and date of when a data collection task was executed, and phone model and make, are also recorded. These data are stored on servers owned and managed by Malmö University in Sweden, they are encrypted during the transmission and at rest and are accessed only by researchers under strict rules.

For peer review only

The GENEActiv actigraphy device stores acceleration data internally. Upon the study's completion, participants return the device, the researcher charges it, extracts and saves the data on the study computer marked with the patient identifier.

Interviews of participants are conducted by researchers in person (when possible) or over the phone and recorded for later analysis with patient's consent.

Malmö University acts as central repository for data, including data collected via Mobistudy and by ASCT. Data is shared among researchers using the Box cloud storage service managed by the University and employing the Cryptomator (https://cryptomator.org/) encryption software.

Collaborators from the University of Edinburgh receive access to pseudonymized data such as general demographic information, MDS-UPDRS assessments, audio data from the vocalization test and the data from the GENEActiv wrist-wearable device. These data are shared through a separate shared folder, containing no personal information.

Data will be removed from the Mobistudy servers upon completion of data collection but retained on researchers' computers during analysis. An archive will be kept for 10 years in accordance with current regulations.

Quality assurance

Data are reviewed monthly to ensure comprehensive and accurate recording at the clinic and through the app. Every two months, interim data analyses are also put in place to allow in-depth quality checks and possible bugs in the software. Non-compliant participants are contacted by the clinical team to explore reasons and propose solutions.

Data analysis plan

Quantitative data will be analyzed using statistical, signal processing and machine learning methods, while qualitative data will be evaluated based on the theoretical foundations underlying the questionnaires and interview questions.

Prediction of symptoms scores using data collected by mobile phones and wearables

From the data collected from activity tests and the wrist-wearable device, we will extract features and train machine learning models for inferring the severity of the disease using the MDS-UPDRS score as a reference. This includes measuring time needed to complete the TUG test, finger dexterity in the FT test, tremor in the HTP and the Drawing tests, and assessing voice distortions in the vocalization test (31).

Data acquired by the GWALK gait analysis device during the TUG tests in clinic will be employed as a reference to validate the algorithms used to analyze the data collected by the app in the same test.

For peer review only

Algorithms will be implemented to assess tremor, bradykinesia, and sleep quality from the actigraphy data obtained from the wearable device. Sleep-related questionnaires will serve as a reference for sleep quality assessment.

Various machine learning classifiers such as AdaBoost, Random Forest (RF), Multi-Layer perceptron (MLP), Support Vector Machine (SVM), XGBoost and similar algorithm for regression analysis will be trained to predict symptoms scores. Regression models will be used to predict the scale of the MDS-UPDRS whereas classifiers will be used to predict scores binned into boarder categories (e.g., no symptoms, low symptoms, high symptoms). A preliminary proof of feasibility has been performed through a regression analysis to infer disease severity (32) based on a dataset with self-reported part of MDS-UPDRS components collected in the mPower study (33). This offered insights into the anticipated quality from data collected in free living conditions. Importantly, a correlation between data collected from smartphones and symptoms' severity was confirmed, but only after removing a substantial number of participants who were considered unreliable. This study aims to address those deficiencies by selectively recruiting participants with clinically diagnosed PD.

Algorithms for classification and regression analysis will be compared on different evaluation metrics including common performance metrics such as accuracy, sensitivity, specificity, and AUC-ROC curve for classifiers. For regression models metrics like mean squared error, mean absolute error, R square measures will be used.

Feature selection (FS) will be performed, and the best predictive features will be identified. Methods mentioned in (34), Relevance, Redundancy, and Complementarity Trade-off (RRCT), mRMR Peng, correlation-based FS, and Recursive Feature Elimination (RFE) will be explored, as well as dimensionality reduction techniques such as Principal Component Analysis (PCA) (35). To assess the performance of different machine learning algorithms k-fold cross validation and leave-one-participant-out cross validation (LOOCV) method will be used, in order to account for participant identity confounding.

Compliance, usability and acceptance

Participants' compliance is measured using activity logs from which we can derive the number of tests performed and questionnaires answered.

Usability and long-term acceptance of the app will be assessed by computing descriptive statistics about the quantitative answers given to the questionnaires and performing a thematic analysis of the interviews based on the theoretical foundations that defined the questions.

Strengths and limitations

Most existing digital systems for PD assessment focus on one symptom e.g., tremor, or cognitive decline. Our proposed approach is multidimensional, aiming at a more holistic view of the participants' status. Another important strength of this study is that sensors are used to measure symptoms objectively and reliably while performing motor tasks and within a daily life environment, thus allowing frequent measurements.

For peer review only

Limitations of the study include: the sample size and sufficiently representing all disease severity levels. Patients with severe PD are excluded from the study, which could impact the generalizability of the results. Furthermore, given the home-based setting of this study, the quality of the data may be affected by the participants' degree of participation and compliance to the study protocol.

Ethics and dissemination

This study was granted ethics approval by the Swedish Ethical Review Authority (Etikprövningsmyndigheten), application number 2022-02885-01.

The results will be reported in peer-reviewed journal and conferences. The results of this study will be shared with the study participants.

Data availability

We aim at publishing an anonymized dataset to enable secondary analysis by other researchers. This anonymized data will be publicly accessible on scientific repositories such as Physionet and Synapse. Anonymization will include removing participants' names and email addresses, reducing detailed demographic information for example by consolidating comorbidities into a singular indicator, eliminating recorded interviews and transcriptions, storing only relative timestamps, and removing identifiable information from responses to interviews and questionnaires.

Conclusions

This study undertakes data collection from participants with PD across different stages of the disease. Data is collected through multiple tasks including questionnaires and activity tests. The aim is to use these data to develop a comprehensive, multi-modal PD symptoms quantifier. Furthermore, participants' feedback will be utilized to assess usability and acceptance of technologies such as smartphones and wearables devices. Ultimately, this study aims to assess the feasibility of assessing PD from home environments, providing clinicians with objective, frequent data for better informed evaluation of patients.

Author contributions

GY drafted the paper which then was critically reviewed and revised by all co-authors. GY, DS, CMO took part on designing the study. AT advised on the methods we will be using for data analysis. PS is the study guarantor. The data collection system was set up by GY and DS. MW and PS are recruiting the participants. The paper was approved by all the authors in the final version of the manuscript.

Funding

This work was supported by Mats Paulsson foundation as part of the grant with project title: Parkapp. Internet of Things and People (IOTAP) research center at Malmö University.

For peer review only

Acknowledgements

We would like to thank our collaborators at Region Stockholm, Karolinska Institute and University of Edinburgh. We would like to thank and acknowledge for the financial support provided by Mats Paulsson foundation and Malmö University. Most importantly, we would like to thank our participants, who have given their time and share their valuable experiences in being part of this study. Finally, we would like to thank the Internet of Things and People research center for their technical and financial support.

Competing interests

None.

For peer review only

Appendix

Daily sleep questionnaire.

Intro:
The following questions are related to the quality of your sleep last night. The answers provided here will be compared with the activity measured by the activity band.

Questions:
1) At what time did you go to sleep yesterday?

2) At what time did you wake up?

3) How did you perceive the quality of sleep last night?
Excellent - Good - Fair – Poor

4) Was falling asleep difficult?
No - Somewhat - Fairly – Very

5) Did you lie awake for long periods during the night?
No - Some - Fairly much - Very much

6) Did you suffer from interruptions?
No - Some - Fairly much - Very muc

For peer review only

Bibliography

1. Organization WH. Neurological Disorders: Public Health Challenges. World Health Organization; 2006. 233 p.

2. Krishnamurthi RV, Ikeda T, Feigin VL. Global, Regional and Country-Specific Burden of Ischaemic Stroke, Intracerebral Haemorrhage and Subarachnoid Haemorrhage: A Systematic Analysis of the Global Burden of Disease Study 2017. Neuroepidemiology. 2020;54(2):171–9.

3. Armstrong MJ, Okun MS. Diagnosis and Treatment of Parkinson Disease: A Review. JAMA. 2020 Feb;323(6):548–60.

4. Bhidayasiri R, Tarsy D. Parkinson's Disease: Hoehn and Yahr Scale. In: Bhidayasiri R, Tarsy D, editors. Movement Disorders: A Video Atlas: A Video Atlas [Internet]. Totowa, NJ: Humana Press; 2012 [cited 2023 Jul 11]. p. 4–5. (Current Clinical Neurology). Available from: https://doi.org/10.1007/978-1-60327-426-5_2

5. Goetz CG, Tilley BC, Shaftman SR, Stebbins GT, Fahn S, Martinez-Martin P, et al. Movement Disorder Society-sponsored revision of the Unified Parkinson's Disease Rating Scale (MDS-UPDRS): Scale presentation and clinimetric testing results: MDS-UPDRS: Clinimetric Assessment. Mov Disord. 2008 Nov 15;23(15):2129–70.

6. Hendricks RM, Khasawneh MT. An Investigation into the Use and Meaning of Parkinson's Disease Clinical Scale Scores. Teive H, editor. Parkinson's Disease. 2021 May 29;2021:1–7.

7. Papapetropoulos S (Spyros). Patient Diaries As a Clinical Endpoint in Parkinson's Disease Clinical Trials. CNS Neuroscience & Therapeutics. 2012;18(5):380–7.

8. Tsanas A, Saunders KEA, Bilderbeck AC, Palmius N, Osipov M, Clifford GD, et al. Daily longitudinal self-monitoring of mood variability in bipolar disorder and borderline personality disorder. Journal of Affective Disorders. 2016 Nov 15;205:225–33.

9. Woodward K, Kanjo E, Brown D, McGinnity TM, Inkster B, Macintyre DJ, et al. Beyond Mobile Apps: A Survey of Technologies for Mental Well-being [Internet]. arXiv; 2020 [cited 2023 May 5]. Available from: http://arxiv.org/abs/1905.00288

10. Lieber B, Taylor BES, Appelboom G, McKhann G, Connolly ES. Motion Sensors to Assess and Monitor Medical and Surgical Management of Parkinson Disease. World Neurosurgery. 2015 Aug 1;84(2):561–6.

11. Linares-del Rey M, Vela-Desojo L, Cano-de la Cuerda R. Mobile phone applications in Parkinson's disease: a systematic review. Neurología (English Edition). 2019 Jan 1;34(1):38–54.

For peer review only

12. Hubble RP, Naughton GA, Silburn PA, Cole MH. Wearable Sensor Use for Assessing Standing Balance and Walking Stability in People with Parkinson's Disease: A Systematic Review. PLOS ONE. 2015 Apr 20;10(4):e0123705.

13. López-Blanco R, Velasco MA, Méndez-Guerrero A, Romero JP, del Castillo MD, Serrano JI, et al. Smartwatch for the analysis of rest tremor in patients with Parkinson's disease. Journal of the Neurological Sciences. 2019 Jun 15;401:37–42.

14. Czech M, Demanuele C, Erb MK, Ramos V, Zhang H, Ho B, et al. The Impact of Reducing the Number of Wearable Devices on Measuring Gait in Parkinson Disease: Noninterventional Exploratory Study. JMIR Rehabilitation and Assistive Technologies. 2020;7(2):e17986.

15. Aghanavesi S, Nyholm D, Senek M, Bergquist F, Memedi M. A smartphone-based system to quantify dexterity in Parkinson's disease patients. Informatics in Medicine Unlocked. 2017 Jan 1;9:11–7.

16. Omberg L, Chaibub Neto E, Perumal TM, Pratap A, Tediarjo A, Adams J, et al. Remote smartphone monitoring of Parkinson's disease and individual response to therapy. Nat Biotechnol. 2021 Aug 9;1–8.

17. Stamate C, Magoulas GD, Kueppers S, Nomikou E, Daskalopoulos I, Jha A, et al. The cloudUPDRS app: A medical device for the clinical assessment of Parkinson's Disease. Pervasive and Mobile Computing. 2018 Jan;43:146–66.

18. Heldman DA, Harris DA, Felong T, Andrzejewski KL, Dorsey ER, Giuffrida JP, et al. Telehealth Management of Parkinson's Disease Using Wearable Sensors: An Exploratory Study. DIB. 2017;1(1):43–51.

19. Wilkinson JR, Spindler M, Wood SM, Marcus SC, Weintraub D, Morley JF, et al. High patient satisfaction with telehealth in Parkinson disease. Neurol Clin Pract. 2016 Jun;6(3):241–51.

20. Cancela J, Pastorino M, Arredondo MT, Hurtado O. A telehealth system for Parkinson's disease remote monitoring. The PERFORM approach. In: 2013 35th Annual International Conference of the IEEE Engineering in Medicine and Biology Society (EMBC). 2013. p. 7492–5.

21. Salvi D, Olsson CM, Ymeri G, Carrasco-Lopez C, Tsang KCH, Shah SA. Mobistudy: mobile-based, platform-independent, multi-dimensional data collection for clinical studies. In: 11th International Conference on the Internet of Things [Internet]. St.Gallen Switzerland: ACM; 2021 [cited 2023 Feb 8]. p. 219–22. Available from: https://dl.acm.org/doi/10.1145/3494322.3494363

22. Beck Depression Inventory–II [Internet]. [cited 2023 Apr 13]. Available from: https://psycnet.apa.org/doiLanding?doi=10.1037%2Ft00742-000

For peer review only

23. Trenkwalder C, Kohnen R, Högl B, Metta V, Sixel-Döring F, Frauscher B, et al. Parkinson's disease sleep scale—validation of the revised version PDSS-2. Movement Disorders. 2011;26(4):644–52.

24. Chaudhuri KR, Martinez-Martin P, Schapira AHV, Stocchi F, Sethi K, Odin P, et al. International multicenter pilot study of the first comprehensive self-completed nonmotor symptoms questionnaire for Parkinson's disease: The NMSQuest study. Movement Disorders. 2006;21(7):916–23.

25. The PDQ-8: Development and validation of a short-form parkinson's disease questionnaire: Psychology & Health: Vol 12, No 6 [Internet]. [cited 2023 Mar 2]. Available from: https://www.tandfonline.com/doi/abs/10.1080/08870449708406741

26. Ohayon M, Wickwire EM, Hirshkowitz M, Albert SM, Avidan A, Daly FJ, et al. National Sleep Foundation's sleep quality recommendations: first report. Sleep Health. 2017 Feb 1;3(1):6–19.

27. Stoyanov SR, Hides L, Kavanagh DJ, Wilson H. Development and Validation of the User Version of the Mobile Application Rating Scale (uMARS). JMIR mHealth and uHealth. 2016 Jun 10;4(2):e5849.

28. Rajak M, Shaw K. An extension of technology acceptance model for mHealth user adoption. Technology in Society. 2021 Nov 1;67:101800.

29. Tsanas A. Investigating Wrist-Based Acceleration Summary Measures across Different Sample Rates towards 24-Hour Physical Activity and Sleep Profile Assessment. Sensors. 2022 Jan;22(16):6152.

30. MacKay DJC. Information Theory, Inference, and Learning Algorithms.

31. Tsanas A, Little MA, McSharry PE, Spielman J, Ramig LO. Novel Speech Signal Processing Algorithms for High-Accuracy Classification of Parkinson's Disease. IEEE Transactions on Biomedical Engineering. 2012 May;59(5):1264–71.

32. Ymeri G, Salvi D, Olsson CM. Linking Data Collected from Mobile Phones with Symptoms Level in Parkinson's Disease: Data Exploration of the mPower Study. In: Tsanas A, Triantafyllidis A, editors. Pervasive Computing Technologies for Healthcare. Cham: Springer Nature Switzerland; 2023. p. 439–49. (Lecture Notes of the Institute for Computer Sciences, Social Informatics and Telecommunications Engineering).

33. Bot BM, Suver C, Neto EC, Kellen M, Klein A, Bare C, et al. The mPower study, Parkinson disease mobile data collected using ResearchKit. Scientific Data. 2016 Mar 3;3(1):160011.

34. Tsanas A. Relevance, redundancy, and complementarity trade-off (RRCT): A principled, generic, robust feature-selection tool. Patterns. 2022 May 13;3(5):100471.

35. Palmerini L, Mellone S, Rocchi L, Chiari L. Dimensionality reduction for the quantitative evaluation of a smartphone-based Timed Up and Go test. In: 2011 Annual

For peer review only

International Conference of the IEEE Engineering in Medicine and Biology Society. 2011. p. 7179–82.

For peer review only

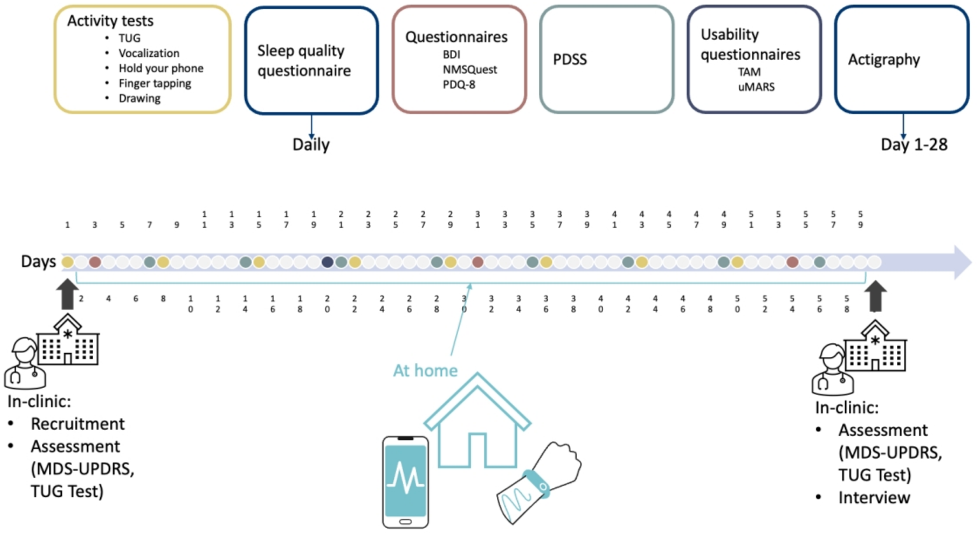

Timeline of the study depicting assessments, tests and questionnaires, when and where they take place.

338x190mm (120 x 120 DPI)

BMJ Open

Quantifying Parkinson's disease severity using mobile wearable devices and machine learning; The ParkApp pilot study protocol

Journal:	*BMJ Open*
Manuscript ID	bmjopen-2023-077766.R1
Article Type:	Protocol
Date Submitted by the Author:	24-Nov-2023
Complete List of Authors:	Ymeri, Gent; Malmo universitet, DVMT<br>Salvi, Dario; Malmö University, DVMT<br>Olsson, Carl Magnus; Malmö universitet, DVMT<br>Wassenburg, Myrthe; Karolinska Institute, Department of Clinical Neuroscience; Academic Specialist Center Torsplan<br>Tsanas, Athanasios; University of Edinburgh<br>Svenningsson, Per; Karolinska Institute, Department of Clinical Neuroscience; Academic Specialist Center Torsplan
**Primary Subject Heading**:	Health informatics
Secondary Subject Heading:	Neurology
Keywords:	Parkinson-s disease < NEUROLOGY, Health informatics < BIOTECHNOLOGY & BIOINFORMATICS, Telemedicine < BIOTECHNOLOGY & BIOINFORMATICS

SCHOLARONE™
Manuscripts

Quantifying Parkinson's disease severity using mobile wearable devices and machine learning; The ParkApp pilot study protocol

Gent Ymeri 1, Dario Salvi 1, Carl Magnus Olsson 1, Myrthe Wassenburg 2,3, Athanasios Tsanas 4,5, and Per Svenningsson 2,3

1 Internet of Things and People research center, Department of Computer Science and Media Technology (DVMT), Malmö University, Sweden
2 Department of Clinical Neuroscience, Karolinska Institute, Sweden
3 Center for Neurology, Academic Specialist Center Torsplan, Region Stockholm, Sweden
4 Usher Institute, Edinburgh Medical School, The University of Edinburgh, United Kingdom
5 Alan Turing Institute, London, United Kingdom

1 firstname.lastname@mau.se
2, 3 firstname.lastname@ki.se
4, 5 firstname.lastname@ed.ac.uk

Corresponding author:
Gent Ymeri; Bassänggatan 2, 211 19, Malmö, Sweden; gent.ymeri@mau.se; +46 40-665 71 71

For peer review only

Abstract

Introduction: the clinical assessment of Parkinson's disease (PD) symptoms can present reliability issues and, with visits typically spaced apart 6 months, can hardly capture their frequent variability. Smartphones and smartwatches along with signal processing and machine learning can facilitate frequent, remote, reliable, and objective assessments of PD from patients' homes.

Aim: To investigate the feasibility, compliance, and user experience of passively and actively measuring symptoms from home environments using data from sensors embedded in smartphones and a wrist-wearable device.

Methods and analysis: In an ongoing clinical feasibility study, participants with a confirmed PD diagnosis are being recruited. Participants perform activity tests, including Timed Up and Go (TUG), tremor, finger tapping, drawing, and vocalization, once a week for two months using the Mobistudy smartphone app in their homes. Concurrently, participants wear the

GENEActiv wrist device for 28 days to measure actigraphy continuously. In addition to using sensors, participants complete the Beck's Depression Inventory, NMSQuest and PDQ-8 questionnaires at baseline, at 1 month and at the end of the study. Sleep disorders are assessed through the Parkinson's Disease Sleep Scale-2 questionnaire (weekly) and a custom sleep quality daily questionnaire. User experience questionnaires, TAM and uMARS are delivered at 1 month. Clinical assessment (MDS-UPDRS) is performed at enrollment and the two-month follow-up visit. During visits, a TUG test is performed using the smartphone and the G-Walk motion sensor as reference device.

Signal processing and machine learning techniques will be employed to analyze the data collected from Mobistudy app and the GENEActiv and correlate them with the MDS-UPDRS. Compliance and user aspects will be informing the long-term feasibility.

Ethics and Dissemination: The study received ethical approval by the Swedish Ethical Review Authority (Etikprövningsmyndigheten), with application number (2022-02885-01). Results will be reported in peer-reviewed journals and conferences. Results will be shared with the study participants.

Keywords: Parkinson’s Disease, Health Informatics, Telemedicine

Wordcount: 3421

Strengths and limitations of this study

- This study utilizes a mobile and wearable device to conveniently, non-invasively, passively, and actively quantify symptoms at home in a user-friendly manner.
- The study combines objective data collected across multiple tests, to evaluate the frequency and severity of motor and non-motor symptoms.
- The study aims at identifying the link between data collected at home and conventional clinical scales, potentially enhancing the remote monitoring and management of Parkinson's disease.
- This study excludes participants with severe symptoms who are unable to use smartphones, which may result in a less diverse sample and limit the generalizability of the findings.
- Data analysis is dependent on the quantity and variability of collected data; success of the study relies on participants’ compliance with the intensive data collection regime.

Introduction

Parkinson’s disease (PD) is a heterogeneous neurodegenerative disorder characterized by third cardinal symptoms: bradykinesia, resting tremor and musculoskeletal rigidity as well as several non-motor symptoms including cognitive alterations, anxiety, depression, hallucinations, pain, speech issues, sleep disorders along with autonomic disorders such as bladder and bowel problems and orthostatic hypotension (1). PD is the second most common neurodegenerative disorder, with a worldwide prevalence estimated to reach 13 million cases by 2040 (2). The diagnosis and assessment of PD are mainly based on clinical findings such as history and physical examinations (3). The most used and recommended

For peer review only

rating scales to measure PD disease severity, progression and functional disability are the Hoehn and Yahr (H&Y) staging scale and the MDS-Unified Parkinson Disease Rating Scale (MDS-UPDRS)(4,5). The MDS-UPDRS is usually performed twice a year during clinic appointments to track disease progression. Both scales can present reliability issues, use ordinal scores and do not track disease burden in a home environment at regular intervals (6).
To complement in-clinic examinations, patients may be asked to record their symptoms in diaries at home; however, these diaries often lack the necessary accuracy (7) needed to effectively supplement clinical assessments and are prone to recall bias. Smartphones offer a more convenient alternative to pen and paper, allowing for more efficiency and accuracy while minimizing such biases (8). Nevertheless, self-reports alone are insufficient to capture the full extent of symptoms, and additional technologies have been demonstrated to provide significant benefits across a range of applications (9). Consequently, there is a need for systems that allow PD patients to be conveniently, reliably, objectively, and longitudinally monitored in their home environments.

Accelerometers, particularly those embedded in mobile phones and smartwatches, have recently been explored as cost-effective, user-friendly solutions to monitor PD symptoms, and facilitating better follow-up, care and improved patient management (10–12). As an example, Lopez-Blanco and co-workers (13) explore the gyroscope embedded in smartwatches to analyze rest tremor in PD patients. Their results show that utilizing smartwatches for assessing rest tremor can correlate with clinical score and is well accepted from users. Czech and co-workers (14) investigate wearable devices to measure gait in PD. Their results prove that measuring gait in PD can be performed reliably with a single accelerometer. However, their study uses a lumbar-mounted accelerometer and does not investigate common wearable devices such as smartwatches or smartphones. Some research projects extend the range of monitored symptoms, such as in (15) where the authors investigate the quantification of dexterity in PD through a smartphone-based system. In this paper, authors incorporate finger tapping and drawing tests in a clinical trial including 19 PD participants and 22 healthy controls. Their results show weak to moderate correlations between UPDRS items and smartphone-based ratings; however, this study was performed in a clinical environment and does not show that the same quality of data can be obtained at home. In addition, the more recent mPower study (16) explores assessment of PD utilizing a smartphone application in home environments. Recruitment of participants in this study was performed online which did not guarantee that all the participants were clinically diagnosed with PD. This study incorporates a number of tasks to gather data with the participants' mobile phones, including finger tapping, vocalization exercises, walk, balance and memory tests. Their results show that the finger tapping task was more predictive for the self-reported status of PD. A smartphone app, cloudUPDRS app, is also introduced in (17) which also includes a number of data collection activities such as reaction, tremor and walking tests, and a wellbeing test, but excluding voice or cognition tests.

Despite the existence of various digital health tools for PD monitoring, these typically focus on a single measurement modality, or the recruitment of the participants does not generalize the results for at-home assessments. A multimodal, user-friendly, and affordable

For peer review only

solution for home-based continuous monitoring of PD could provide significant advantages for both patients and clinicians.

Considering this, the primary aim of this study is to determine the feasibility of reliably measuring PD symptoms using mobile phones and wearable devices at home and to provide a more holistic overview of symptomatology compared to what has been attempted in previous studies. The study is supported by the development of a novel, multi-modal symptoms tracker system, which both passively and actively quantifies motor symptoms such as bradykinesia, gait impairment, tremor, voice disorders, as well as non-motor symptoms like depression, and sleep disturbances.

Methods and Analysis

Study design and participants

This clinical feasibility study involves the recruitment of 30 participants with clinically diagnosed PD at the Center for neurology, Academic Specialist Center Torsplan (ASCT), Stockholm Health Services, Region Stockholm.

Participants are recruited based on the inclusion and exclusion criteria summarized in Table 1. Guided by similar studies that have used between 10-50 participants (18–20), the goal of this study is towards hypothesis generation and assessing feasibility. Efforts are made to recruit participants with a diverse range of PD symptoms while avoiding over-representation of one sex or age category. Given the limited number of participants and the exploratory nature of this study, no specific software is used for randomization; an ad-hoc approach is adopted depending on the circumstances. Recruitment started in the fourth quarter of 2022, with plans to complete data collection in the fourth quarter of 2023.

Table 1: Inclusion/ exclusion criteria

Inclusion criteria	*Exclusion criteria*
Must be diagnosed with Parkinson's Disease and under treatment at Karolinska University Hospital or Academic Specialist Center Torsplan	Not able to use a smartphone independently
Must have mild or moderate motor and non-motor symptoms	Unable to read and understand Swedish
Must own a smartphone, Android or iPhone	Severe symptoms (H&Y 3 and above)

Outcomes

The research questions that this study addresses are:

A. Are the multimodal measurements collected by mobile phones and wearable devices valid for estimating Parkinson's disease symptoms?
B. What extracted information/ features from raw data are predictive of MDS-UPDRS scores using data collected by mobile phones and wearables?
C. Are mobile phones and wearable devices usable and accepted for data collection?

For peer review only

These research questions entail collecting data from devices at participants' home and reference data in-clinic and developing algorithms to associate the MDS-UPDRS score from the data collected at home. The questions also imply measuring compliance to the data regime protocol, usability of the technology and long-term acceptance.

Patient and Public Involvement

None.

Data collection

Data from participants are collected through two clinic visits - at baseline and after two months - as well as in between visits by using a mobile phone app and a wearable device in home environment. The mobile phone app used in this study is Mobistudy (21), a free and open-source app designed for clinical research. The app prompts users to perform scheduled tasks during their two-month participation in the study, as outlined in Table 2. Tasks include answering questionnaires and performing motor tests. The motor tests are prompted to be performed twice: once prior to medication intake and again post-intake to measure ON and OFF states. While there is no designated time of day for these tests, participants are instructed to consistently follow this pre- and post-medication schedule.

The tasks included in the Mobistudy app for this study are:

- Questionnaires:
    - Beck's depression inventory-II (BDI-II) (22). This is used to quantify depression.
    - Parkinson's disease sleep scale (PDSS-2) (23). Used to quantify sleep disorder.
    - NMSQuest (24). Measures non-motor symptoms.
    - PDQ-8 (25). Short scale to assess motor and non-motor symptoms in daily life.
    - Sleep quality questionnaire. Based on (26), is a custom daily short questionnaire to assess sleep quality (full questions list in Appendix 1).
    - User Version of the Mobile Application Rating Scale (uMARS) (27). Measures usability and the quality of the app.
    - Technology Acceptance Model (TAM) (28). Estimates long-term usage of the app.
- Timed up and go test (TUG test): Participants are instructed to stand up from a chair, walk 3 meters, turn around, return to the chair, and sit down. The mobile phone, secured in a waist belt around the hip, records data from inertial sensors (accelerometer and gyroscope) and estimates the total time required to complete the task.
- Vocalization test: Participants record their voice during a vocalization exercise guided by the app. The exercise consists of pronouncing the sustained vowels “a”, “i” and “u” for as long as possible.

For peer review only

- Hold the phone test (HTP test): Participants are instructed to hold the phone for 60 seconds while the phone records inertial sensors data (acceleration and orientation). The task is repeated for each hand in three positions: resting the hand holding the phone in the lap while sitting, extending the arm at shoulder's height, and moving the arm from outstretched to touching the nose repeatedly.
- Finger tapping test (FT test): Participants are instructed to rapidly tap two buttons on the screen using the index and middle fingers.
- Drawing test: Participants are instructed to draw a square and a squared spiral on the screen by tracing a marked line with their index finger.

In addition to the app, a wrist-worn actigraphy device (GENEActiv, Activinsights, UK) is used to collect continuous three-dimensional acceleration data in free-living conditions. A gait analysis device (GWALK, BTS Bioengineering, Italy) is used to gather reference gait analysis data during the TUG test in clinic, at enrolment and conclusion of the study. The GENEActiv device is set to capture data at a sample rate of 25Hz. Whilst 10Hz is considered sufficient for wrist-based acceleration in day-to-day activities (29), this study adheres to the Nyquist theorem by using at least twice the maximum frequency of tremor (30).

While aiming at including a wide range of symptomatology, in this first feasibility study, we decided to focus on motor symptoms, also given the nature of the sensors employed. We decided to exclude cognitive function, as this would have required the implementation of additional interactive features on the Mobistudy app that were not justified by the scope of the research.

Participants are approached during their regular visits at the ASCT clinic. If they give informed consent to participate, they receive an introduction to the study, instructions, a waist belt, and an actigraphy device. The Mobistudy app is downloaded and installed on their smartphone, and participants create an account. A series of demo tests using Mobistudy is available for participants to practice and ensure understanding before the study starts. Each test includes in-app instructions. A TUG test, performed with the GWALK gait analysis reference device, and a full MDS-UPDRS assessment by a neurologist, are conducted during enrollment.

During the eight-week at-home period, participants complete tests and answer questionnaires using their phones and the Mobistudy app (illustrated in Figure 1). Participants are contacted on day ten and on day 30 either via email or phone call to check compliance and the presence of any technical or practical issues.

Upon completing of the study, participants attend a second clinic visit, during which they perform a TUG test with GWALK, undergo a full MDS-UPDRS assessment, and complete an interview. The semi-structured interview is designed to complement questions asked in the usability questionnaires and covers motivations for participation, usability, user friendliness, acceptance, perceptions on trust, and suggestions for improvements (full schedule in Appendix 2). A researcher conducts the interview with the participant either in the clinic or over the phone if consent is granted. Interviews are expected to last no more than 30 minutes and are audio recorded.

For peer review only

Table 2: The schedule for when activity tasks and questionnaires are performed by the recruited participants.

Activity tasks and Questionnaires	*Day after recruitment*							
Timed-up-and-go-test	1	8	15	22	29	36	43	50
Beck's depression inventory	3	31	54					
Parkinson's disease sleep scale	7	14	21	28	35	42	49	56
NMSQuest	3	31	54					
PDQ-8	3	31	54					
Vocalization test	1	8	15	22	29	36	43	50
Hold your phone test	1	8	15	22	29	36	43	50
Finger tapping test	1	8	15	22	29	36	43	50
Drawing test	1	8	15	22	29	36	43	50
Sleep quality questionnaire	Daily							
uMARS	20							
TAM	20							
Actigraphy	Continuously, recording at 25Hz until battery is depleted after 28 days							

Data Management

Clinical data, including patients' demographics (age, sex, year of diagnosis of PD, year of onset of first symptoms, deep brain stimulation device status, any PD-related surgery, medication regime and comorbidities) as well as in-clinic results (evaluated MDS-UPDRS and TUG test), are collected by the ASCT. These data are stored on an encrypted disk on a computer managed by the research team.

App-based data is collected using the Mobistudy platform (21). This includes participants' profiles - name, surname, date of birth, sex, height, weight, long-term diseases, long-term medications-, questionnaire responses and sensors data. Sensor's data includes acceleration and orientation during the TUG test and HTP test, voice recordings collected during the vocalization test, timestamps of taps during the finger tapping test and finger coordinates during the drawing test. Metadata including time and date of when a data collection task was executed, and phone model and make, are also recorded. These data are stored on servers owned and managed by Malmö University in Sweden, they are encrypted during the transmission and at rest and are accessed only by researchers under strict rules.

The GENEActiv actigraphy device stores acceleration data internally. Upon the study's completion, participants return the device, the researcher charges it, extracts and saves the data on the study computer marked with the patient identifier.

Interviews of participants are conducted by researchers in person (when possible) or over the phone and recorded for later analysis with patient's consent.

Malmö University acts as central repository for data, including data collected via Mobistudy and by ASCT. Data is shared among researchers using the Box cloud storage service

For peer review only

managed by the University and employing the Cryptomator (https://cryptomator.org/) encryption software.

Collaborators from the University of Edinburgh receive access to pseudonymized data such as general demographic information, MDS-UPDRS assessments, audio data from the vocalization test and the data from the GENEActiv wrist-wearable device. These data are shared through a separate shared folder, containing no personal information.

Data will be removed from the Mobistudy servers upon completion of data collection but retained on researchers' computers during analysis. An archive will be kept for 10 years in accordance with current regulations.

Quality assurance

Data are reviewed monthly to ensure comprehensive and accurate recording at the clinic and through the app. Every two months, interim data analyses are also put in place to allow in-depth quality checks and possible bugs in the software. Non-compliant participants are contacted by the clinical team to explore reasons and propose solutions.

Data analysis plan

Quantitative data will be analyzed using statistical, signal processing and machine learning methods, while qualitative data will be evaluated based on the theoretical foundations underlying the questionnaires and interview questions.

Validity of measurements collected by mobile phones and wearables

From the data collected from activity tests and the wrist-wearable device, we will extract features and train machine learning models for inferring the severity of the disease using the MDS-UPDRS score as a reference. This includes measuring time needed to complete the TUG test, finger dexterity in the FT test, tremor in the HTP and the Drawing tests, and assessing voice distortions in the vocalization test (31). The main metric for assessing validity will be correlation between features and/or inferred scores and actual scores. We will associate the measurements to the MDS-UPDRS closest in time. The variability of fluctuating symptoms we will be addressed by exploring how averaging features, including before and after medication and over the weeks, affects the correlation. The data collected by the wearable will also offer a reference to understand if and in what way symptoms have evolved over time.

As targets, we will employ both summative scores, for example for Part 3 (motor symptoms), which will be compared with aggregated results from all the tests, and specific items, when those can me mapped to the result of a specific test, for example: finger tapping with item 3.4.

Data acquired by the GWALK gait analysis device during the TUG tests in clinic will be employed as a reference to validate the algorithms used to analyze the data collected by the app in the same test.

For peer review only

Algorithms will be implemented to assess tremor, bradykinesia, and sleep quality from the actigraphy data obtained from the wearable device. Sleep-related questionnaires will serve as a reference for sleep quality assessment.

Various machine learning classifiers such as AdaBoost, Random Forest (RF), Multi-Layer perceptron (MLP), Support Vector Machine (SVM), XGBoost and similar algorithm for regression analysis will be trained to predict symptoms scores. Regression models will be used to predict the scale of the MDS-UPDRS whereas classifiers will be used to predict scores binned into boarder categories (e.g., no symptoms, low symptoms, high symptoms). A preliminary proof of feasibility has been performed through a regression analysis to infer disease severity (32) based on a dataset with self-reported part of MDS-UPDRS components collected in the mPower study (33). This offered insights into the anticipated quality from data collected in free living conditions. Importantly, a correlation between data collected from smartphones and symptoms' severity was confirmed, but only after removing a substantial number of participants who were considered unreliable. This study aims to address those deficiencies by selectively recruiting participants with clinically diagnosed PD.

Algorithms for classification and regression analysis will be compared on different evaluation metrics including common performance metrics such as accuracy, sensitivity, specificity, and AUC-ROC curve for classifiers. For regression models metrics like mean squared error, mean absolute error, R square measures will be used.

Feature selection (FS) will be performed, and the best predictive features will be identified. Methods mentioned in (34), Relevance, Redundancy, and Complementarity Trade-off (RRCT), mRMR Peng, correlation-based FS, and Recursive Feature Elimination (RFE) will be explored, as well as dimensionality reduction techniques such as Principal Component Analysis (PCA) (35). To assess the performance of different machine learning algorithms k-fold cross validation and leave-one-participant-out cross validation (LOOCV) method will be used, in order to account for participant identity confounding.

Compliance, usability and acceptance

Participants' compliance is measured using activity logs from which we can derive the number of tests performed and questionnaires answered.

Usability and long-term acceptance of the app will be assessed by computing descriptive statistics about the quantitative answers given to the questionnaires and performing a thematic analysis of the interviews based on the theoretical foundations that defined the questions.

Strengths and limitations

Most existing digital systems for PD assessment focus on one symptom e.g., tremor, or cognitive decline. Our proposed approach is multidimensional, aiming at a more holistic view of the participants' status. Another important strength of this study is that sensors are used to measure symptoms objectively and reliably while performing motor tasks and within a daily life environment, thus allowing frequent measurements.

For peer review only

Limitations of the study include: the sample size and sufficiently representing all disease severity levels. Patients with severe PD are excluded from the study, which could impact the generalizability of the results. Furthermore, given the home-based setting of this study, the quality of the data may be affected by the participants' degree of participation and compliance to the study protocol.

Ethics and dissemination

This study was granted ethics approval by the Swedish Ethical Review Authority (Etikprövningsmyndigheten), application number 2022-02885-01.

The results will be reported in peer-reviewed journal and conferences. The results of this study will be shared with the study participants.

Data availability

We aim at publishing an anonymized dataset to enable secondary analysis by other researchers. This anonymized data will be publicly accessible on scientific repositories such as Physionet and Synapse. Anonymization will include removing participants' names and email addresses, reducing detailed demographic information for example by consolidating comorbidities into a singular indicator, eliminating recorded interviews and transcriptions, storing only relative timestamps, and removing identifiable information from responses to interviews and questionnaires.

Author contributions

GY: Methodology, Software, Data Curation, Formal analysis, Investigation, Writing - Original Draft, Visualization.
DS: Conceptualization, Methodology, Software, Writing - Review & Editing, Supervision, Project administration, Funding acquisition.
CMO: Conceptualization, Methodology, Writing - Review & Editing, Supervision, Funding acquisition.
MW: Investigation, Resources, Data Curation, Writing - Review & Editing.
AT: Conceptualization, Methodology, Writing - Review & Editing, Supervision, Funding acquisition.
PS: Conceptualization, Methodology, Resources, Writing - Review & Editing, Supervision, Funding acquisition.

Funding

Mats Paulsson foundation, grant number: NA.
Internet of Things and People (IOTAP) research center, grant number: NA.
Malmö University, grant number: NA.

Acknowledgements

We would like to thank our collaborators at Region Stockholm, Karolinska Institute and University of Edinburgh. We would like to thank and acknowledge for the financial support provided by Mats Paulsson foundation and Malmö University. Most importantly, we would

For peer review only

like to thank our participants, who have given their time and share their valuable experiences in being part of this study. Finally, we would like to thank the Internet of Things and People research center for their technical and financial support.

Competing interests

None.

For peer review only

Figure 1 Timeline of the study depicting assessments, tests and questionnaires, when and where they take place.

For peer review only

Bibliography

1. Organization WH. Neurological Disorders: Public Health Challenges. World Health Organization; 2006. 233 p.

2. Krishnamurthi RV, Ikeda T, Feigin VL. Global, Regional and Country-Specific Burden of Ischaemic Stroke, Intracerebral Haemorrhage and Subarachnoid Haemorrhage: A Systematic Analysis of the Global Burden of Disease Study 2017. Neuroepidemiology. 2020;54(2):171–9.

3. Armstrong MJ, Okun MS. Diagnosis and Treatment of Parkinson Disease: A Review. JAMA. 2020 Feb;323(6):548–60.

4. Bhidayasiri R, Tarsy D. Parkinson's Disease: Hoehn and Yahr Scale. In: Bhidayasiri R, Tarsy D, editors. Movement Disorders: A Video Atlas: A Video Atlas [Internet]. Totowa, NJ: Humana Press; 2012 [cited 2023 Jul 11]. p. 4–5. (Current Clinical Neurology). Available from: https://doi.org/10.1007/978-1-60327-426-5_2

5. Goetz CG, Tilley BC, Shaftman SR, Stebbins GT, Fahn S, Martinez-Martin P, et al. Movement Disorder Society-sponsored revision of the Unified Parkinson's Disease Rating Scale (MDS-UPDRS): Scale presentation and clinimetric testing results: MDS-UPDRS: Clinimetric Assessment. Mov Disord. 2008 Nov 15;23(15):2129–70.

6. Hendricks RM, Khasawneh MT. An Investigation into the Use and Meaning of Parkinson's Disease Clinical Scale Scores. Teive H, editor. Parkinson's Disease. 2021 May 29;2021:1–7.

7. Papapetropoulos S (Spyros). Patient Diaries As a Clinical Endpoint in Parkinson's Disease Clinical Trials. CNS Neuroscience & Therapeutics. 2012;18(5):380–7.

8. Tsanas A, Saunders KEA, Bilderbeck AC, Palmius N, Osipov M, Clifford GD, et al. Daily longitudinal self-monitoring of mood variability in bipolar disorder and borderline personality disorder. Journal of Affective Disorders. 2016 Nov 15;205:225–33.

9. Woodward K, Kanjo E, Brown D, McGinnity TM, Inkster B, Macintyre DJ, et al. Beyond Mobile Apps: A Survey of Technologies for Mental Well-being [Internet]. arXiv; 2020 [cited 2023 May 5]. Available from: http://arxiv.org/abs/1905.00288

10. Lieber B, Taylor BES, Appelboom G, McKhann G, Connolly ES. Motion Sensors to Assess and Monitor Medical and Surgical Management of Parkinson Disease. World Neurosurgery. 2015 Aug 1;84(2):561–6.

11. Linares-del Rey M, Vela-Desojo L, Cano-de la Cuerda R. Mobile phone applications in Parkinson's disease: a systematic review. Neurología (English Edition). 2019 Jan 1;34(1):38–54.

For peer review only

12. Hubble RP, Naughton GA, Silburn PA, Cole MH. Wearable Sensor Use for Assessing Standing Balance and Walking Stability in People with Parkinson's Disease: A Systematic Review. PLOS ONE. 2015 Apr 20;10(4):e0123705.

13. López-Blanco R, Velasco MA, Méndez-Guerrero A, Romero JP, del Castillo MD, Serrano JI, et al. Smartwatch for the analysis of rest tremor in patients with Parkinson's disease. Journal of the Neurological Sciences. 2019 Jun 15;401:37–42.

14. Czech M, Demanuele C, Erb MK, Ramos V, Zhang H, Ho B, et al. The Impact of Reducing the Number of Wearable Devices on Measuring Gait in Parkinson Disease: Noninterventional Exploratory Study. JMIR Rehabilitation and Assistive Technologies. 2020;7(2):e17986.

15. Aghanavesi S, Nyholm D, Senek M, Bergquist F, Memedi M. A smartphone-based system to quantify dexterity in Parkinson's disease patients. Informatics in Medicine Unlocked. 2017 Jan 1;9:11–7.

16. Omberg L, Chaibub Neto E, Perumal TM, Pratap A, Tediarjo A, Adams J, et al. Remote smartphone monitoring of Parkinson's disease and individual response to therapy. Nat Biotechnol. 2021 Aug 9;1–8.

17. Stamate C, Magoulas GD, Kueppers S, Nomikou E, Daskalopoulos I, Jha A, et al. The cloudUPDRS app: A medical device for the clinical assessment of Parkinson's Disease. Pervasive and Mobile Computing. 2018 Jan;43:146–66.

18. Heldman DA, Harris DA, Felong T, Andrzejewski KL, Dorsey ER, Giuffrida JP, et al. Telehealth Management of Parkinson's Disease Using Wearable Sensors: An Exploratory Study. DIB. 2017;1(1):43–51.

19. Wilkinson JR, Spindler M, Wood SM, Marcus SC, Weintraub D, Morley JF, et al. High patient satisfaction with telehealth in Parkinson disease. Neurol Clin Pract. 2016 Jun;6(3):241–51.

20. Cancela J, Pastorino M, Arredondo MT, Hurtado O. A telehealth system for Parkinson's disease remote monitoring. The PERFORM approach. In: 2013 35th Annual International Conference of the IEEE Engineering in Medicine and Biology Society (EMBC). 2013. p. 7492–5.

21. Salvi D, Olsson CM, Ymeri G, Carrasco-Lopez C, Tsang KCH, Shah SA. Mobistudy: mobile-based, platform-independent, multi-dimensional data collection for clinical studies. In: 11th International Conference on the Internet of Things [Internet]. St.Gallen Switzerland: ACM; 2021 [cited 2023 Feb 8]. p. 219–22. Available from: https://dl.acm.org/doi/10.1145/3494322.3494363

22. Beck Depression Inventory–II [Internet]. [cited 2023 Apr 13]. Available from: https://psycnet.apa.org/doiLanding?doi=10.1037%2Ft00742-000

For peer review only

23. Trenkwalder C, Kohnen R, Högl B, Metta V, Sixel-Döring F, Frauscher B, et al. Parkinson's disease sleep scale—validation of the revised version PDSS-2. Movement Disorders. 2011;26(4):644–52.

24. Chaudhuri KR, Martinez-Martin P, Schapira AHV, Stocchi F, Sethi K, Odin P, et al. International multicenter pilot study of the first comprehensive self-completed nonmotor symptoms questionnaire for Parkinson's disease: The NMSQuest study. Movement Disorders. 2006;21(7):916–23.

25. The PDQ-8: Development and validation of a short-form parkinson's disease questionnaire: Psychology & Health: Vol 12, No 6 [Internet]. [cited 2023 Mar 2]. Available from: https://www.tandfonline.com/doi/abs/10.1080/08870449708406741

26. Ohayon M, Wickwire EM, Hirshkowitz M, Albert SM, Avidan A, Daly FJ, et al. National Sleep Foundation's sleep quality recommendations: first report. Sleep Health. 2017 Feb 1;3(1):6–19.

27. Stoyanov SR, Hides L, Kavanagh DJ, Wilson H. Development and Validation of the User Version of the Mobile Application Rating Scale (uMARS). JMIR mHealth and uHealth. 2016 Jun 10;4(2):e5849.

28. Rajak M, Shaw K. An extension of technology acceptance model for mHealth user adoption. Technology in Society. 2021 Nov 1;67:101800.

29. Tsanas A. Investigating Wrist-Based Acceleration Summary Measures across Different Sample Rates towards 24-Hour Physical Activity and Sleep Profile Assessment. Sensors. 2022 Jan;22(16):6152.

30. MacKay DJC. Information Theory, Inference, and Learning Algorithms.

31. Tsanas A, Little MA, McSharry PE, Spielman J, Ramig LO. Novel Speech Signal Processing Algorithms for High-Accuracy Classification of Parkinson's Disease. IEEE Transactions on Biomedical Engineering. 2012 May;59(5):1264–71.

32. Ymeri G, Salvi D, Olsson CM. Linking Data Collected from Mobile Phones with Symptoms Level in Parkinson's Disease: Data Exploration of the mPower Study. In: Tsanas A, Triantafyllidis A, editors. Pervasive Computing Technologies for Healthcare. Cham: Springer Nature Switzerland; 2023. p. 439–49. (Lecture Notes of the Institute for Computer Sciences, Social Informatics and Telecommunications Engineering).

33. Bot BM, Suver C, Neto EC, Kellen M, Klein A, Bare C, et al. The mPower study, Parkinson disease mobile data collected using ResearchKit. Scientific Data. 2016 Mar 3;3(1):160011.

34. Tsanas A. Relevance, redundancy, and complementarity trade-off (RRCT): A principled, generic, robust feature-selection tool. Patterns. 2022 May 13;3(5):100471.

35. Palmerini L, Mellone S, Rocchi L, Chiari L. Dimensionality reduction for the quantitative evaluation of a smartphone-based Timed Up and Go test. In: 2011 Annual

For peer review only

International Conference of the IEEE Engineering in Medicine and Biology Society. 2011. p. 7179–82.

For peer review only

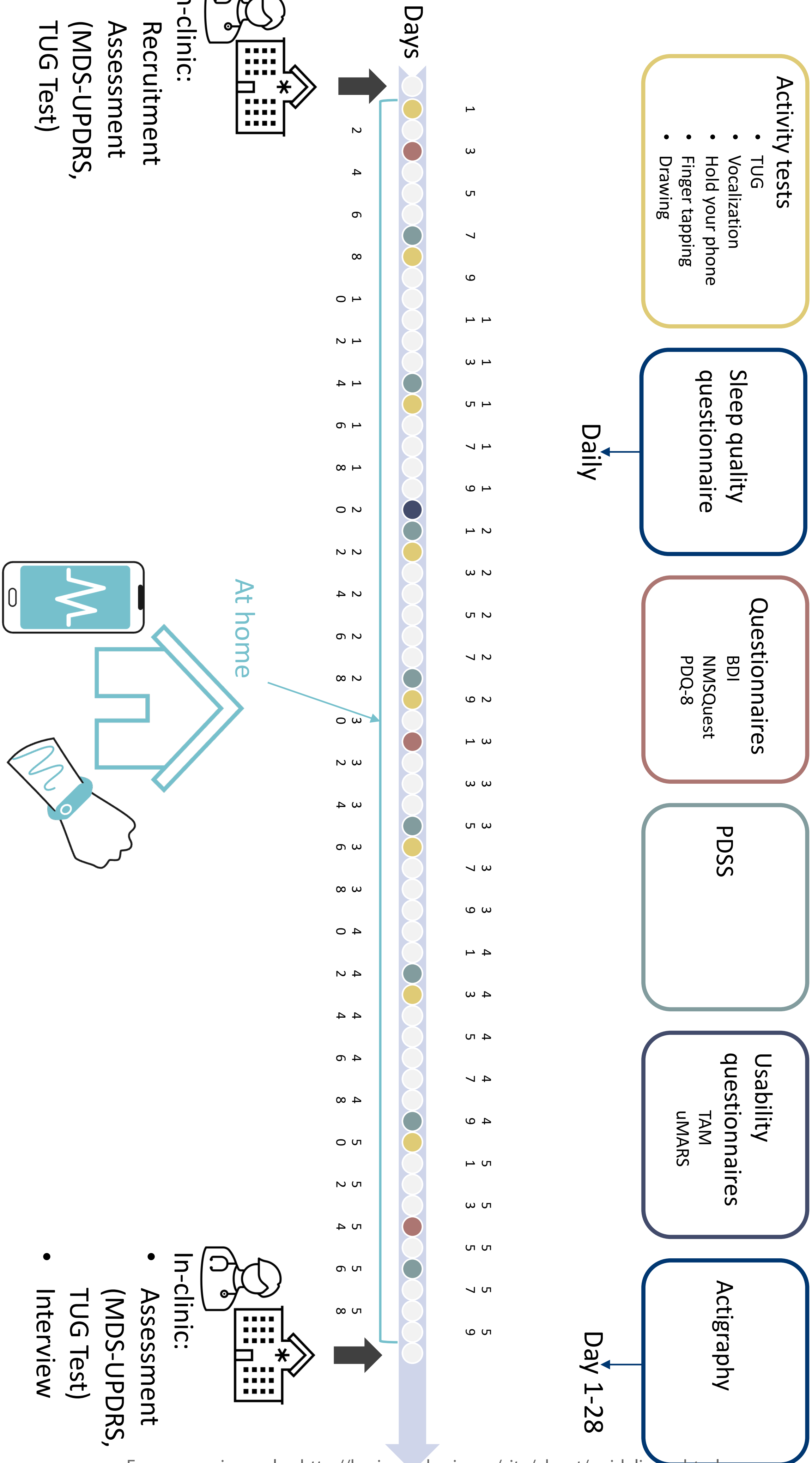
Activity tests
TUG
Vocalization
Hold your phone
Finger tapping
Drawing
Sleep quality questionnaire
Daily
Questionnaires
BDI
NMSQuest
PDQ-8
PDSS
Usability questionnaires
TAM
uMARS
Actigraphy
Day 1-28
Days
In-clinic:
Recruitment
Assessment (MDS-UPDRS, TUG Test)
At home
In-clinic:
Assessment (MDS-UPDRS, TUG Test)
Interview

Appendix 1

Daily sleep questionnaire.

Intro:
The following questions are related to the quality of your sleep last night. The answers provided here will be compared with the activity measured by the activity band.

Questions:
1) At what time did you go to sleep yesterday?

2) At what time did you wake up?

3) How did you perceive the quality of sleep last night?
Excellent - Good - Fair – Poor

4) Was falling asleep difficult?
No - Somewhat - Fairly – Very

5) Did you lie awake for long periods during the night?
No - Some - Fairly much - Very much

6) Did you suffer from interruptions?
No - Some - Fairly much - Very much

For peer review only

Appendix 2

The interview will follow a semi-structured format with questions based on usability, acceptance and trust models. One person will interview the patient either in the clinic or over the phone. Interviews will be audio recorded and notes will be taken. Interviews will last no longer than 30 minutes.
The following are example questions which the interviewers may adapt depending on the context. To keep the interview time limited, 2 questions for theme will be chosen and, depending on the provided answers, more questions may be asked to further elaborate some concepts.

Theme: Participation
1. Why did you join this study?
2. Have you appreciated being part of a research study, or has it been a bother to you? Why do you feel this way?
3. Would you be interested in future participation in research, if there was an opportunity to do so? Why?
4. Would you like to keep using the app to participate in other studies?

Theme: Onboarding, understanding and engagement
1. How long did it take you to start using Mobistudy?
2. Did you encounter any technical issue?
3. Were there parts (e.g. tests or menus) that were hard to know if you did the right thing with? Which? Why?
4. Are there tests (if so, which) that you still have questions on how they work, or do you understand all of them now?
5. Any test that you were particularly interested in the results from? Why?
6. Is there anything we could add or remove from the app to make it more interesting for you?

Theme: Impact (conventional clinical practice vs app use)
1. Are there parts of your life that are affected by your PD which you are not reporting in the traditional clinical meetings?
2. Are there parts of your life that are affected by your PD which you aren't reporting in the Mobistudy app?
3. Have you learned something new about your own PD during the research project? What? How?
4. How does this impact your day-to-day life?
5. Did the use of the app create any concern about your health?
6. Do you think using the app would improve the way your doctors keep your health under control?
7. Would you trust this technology to substitute your periodic appointment with your doctor?

For peer review only

Theme: Trust

1. Do you have any concern about your data being shared with parties that you do not trust?
2. What sort of parties do you consider untrustworthy?
3. Did you have any concern about being "observed" (even if by people you trust)?
4. Can you give me examples of apps you trust?
5. And apps you do not trust?

For peer review only
